# Duck Tembusu virus in North Vietnam: epidemiological and genetic analysis reveals novel virus strains

**DOI:** 10.3389/fvets.2024.1366904

**Published:** 2024-05-14

**Authors:** Hieu Van Dong, Giang Thi Huong Tran, Tra Thi Thu Vu, Ngan Hong Thi Le, Yen Thi Hoang Nguyen, Witsanu Rapichai, Amonpun Rattanasrisomporn, Chaiwat Boonkaewwan, Dao Anh Tran Bui, Jatuporn Rattanasrisomporn

**Affiliations:** ^1^Faculty of Veterinary Medicine, Vietnam National University of Agriculture, Hanoi, Vietnam; ^2^Dak Lak Sub-Department of Livestock Production and Animal Health, Dak Lak, Vietnam; ^3^Department of Companion Animal Clinical Sciences, Faculty of Veterinary Medicine, Kasetsart University, Bangkok, Thailand; ^4^Department of Biochemistry, Faculty of Science, Kasetsart University, Bangkok, Thailand; ^5^Interdisciplinary of Genetic Engineering and Bioinformatics, Graduate School, Kasetsart University, Bangkok, Thailand; ^6^Akkhraratchakumari Veterinary College, Walailak University, Tha Sala District, Thailand

**Keywords:** ducks, genetic characterization, PCR, Tembusu virus, Vietnam

## Abstract

Tembusu virus (TMUV) is an important infectious disease, causing economic losses in duck production. Since the first report of TMUV infection in Vietnam in 2020, the disease has persisted and affected poultry production in the country. This study conducted epidemiological and genetic characterization of the viral strains circulating in north Vietnam based on 130 pooled tissue samples collected in six provinces/cities during 2021. The TMUV genome was examined using conventional PCR. The results indicated that 21 (16.15%) samples and 9 (23.68%) farms were positive for the viral genome. The positive rate was 59.26% for ducks at ages 2–4 weeks, which was significantly higher than for ducks at ages >4 weeks and < 2 weeks. Genetic analysis of the partial envelope gene (891 bp) sequences indicated that the five Vietnamese TMUVs shared 99.55–100% nucleotide identity, while the rates were in the range 99.59–100% based on the pre-membrane gene sequences (498 bp). The five Vietnamese TMUV strains obtained formed a novel single subcluster. These strains were closely related to Chinese strains and differed from the vaccine strain, suggesting that Vietnamese TMUV strains were field viruses. It needs to be further studied on vaccine development to prevent effects of TMUV infection on poultry production across Vietnam.

## 1 Introduction

Duck farming is a long-standing agricultural venture in Asia. However, this sector faces challenges from various infectious agents, including duck Circovirus, Sitiawan virus, and duck Tembusu virus (TMUV) (1–3). TMUV, classified within the genus *Flavivirus* of the Flaviviridae family, is an arthropod-borne virus characterized by a single-stranded, positive-sense RNA genome. It was originally isolated in 1955 from *Culex tritaeniorhynchus* mosquitoes in Kuala Lumpur, Malaysia (3). Nevertheless, its potential implications for human and animal health remain incompletely understood. Within the *Flavivirus* genus, several members, such as West Nile virus (WNV), dengue virus (DENV), yellow fever virus (YFV), Japanese encephalitis virus (JEV), and Zika virus, exhibit zoonotic properties and serve as major vector-borne pathogens responsible for millions of annual infections. These infections can manifest with a spectrum of clinical presentations, ranging from mild febrile symptoms to severe and potentially fatal hemorrhagic or neurologic diseases. This viral genus is characterized by its 30–60 nm icosahedral envelope capsid containing a single-stranded positive-sense RNA genome approximately 11 Kb in length. The genomic organization encoding the singular polyprotein is three structural proteins of capsid (C)-premembrane (prM)-envelope (E) and seven non-structural (NS) proteins (NS1, NS2A, NS2B, NS3, NS4A, NS4B, NS5) that are produced through the actions of viral and cellular proteases. Surrounding the coding region are untranslated regions at both the 5′ and 3′ ends, which form conserved stem-loop structures (3, 4). Primarily, the composition of the viral particle is shaped by the structural proteins, which play a pivotal role in mediating viral fusion with host cells. These proteins are essential in various processes, including binding to virus receptors, facilitating entry, and associating fusion events. In contrast, the non-structural (NS) proteins are primarily engaged in activities, such as viral RNA replication, the assembly of virions, and the evasion of innate immune responses (5).

Within natural ecosystems, avian species are reservoir hosts for a multitude of flaviviruses, such as West Nile virus (WNV) (6), Sitiawan virus (3), Usutu virus (7, 8), and Bagaza virus (9). In April 2010, the causative agent of duck egg-drop disease in China was first recognized as duck TMUV. This ailment is distinguished by a decrease in egg yield, an abrupt reduction in feed intake, and the emergence of neurological manifestations among afflicted egg-laying and breeder ducks (4). Egg-drop disease impacts a diverse array of duck breeds, encompassing both meat-producing and egg-laying categories. This spectrum includes Pekin ducks, Cherry Valley ducks, Shaoxing ducks, Jinyun ducks, Longyan ducks, Jinding ducks, Khaki-Campbell ducks, Muscovy ducks, and domesticated mallards (10). The virus is still circulating and affecting duck production and many countries. It would be further studied on epidemiological and genetic characterization of the viral strains, which gives important information for developing vaccine and preventive strategies.

In Vietnam, the first study on TMUV infection was reported by Dang et al. Those authors used a PCR method to detect the TMUV genome in clinically suspected ducks in Hanoi city. Phylogenetic analysis of the partial NS5B gene sequences suggested that the three Vietnamese TMUV strains were genetically related to the DK/TH/CU-1 strain (KR061333) detected in Thailand (11). The aim of the current study was to carry out further epidemiological and genetic characterization of the TMUV strains circulating in several cities and provinces in north Vietnam based on their partial E and prM gene sequences.

## 2 Methods

### 2.1 Ethics statement

The study did not consist of any studies involving human participants. Samples were collected from ducks farmed in north Vietnam under the auspices of the Vietnam National University of Agriculture and the protocol for sampling purposes was submitted and approved by the Committee on Animal Research and Ethics of the University (CARE-2021/04). Permission was obtained from the duck farm owners before sampling.

### 2.2 Sample collection

**Figure 1:**
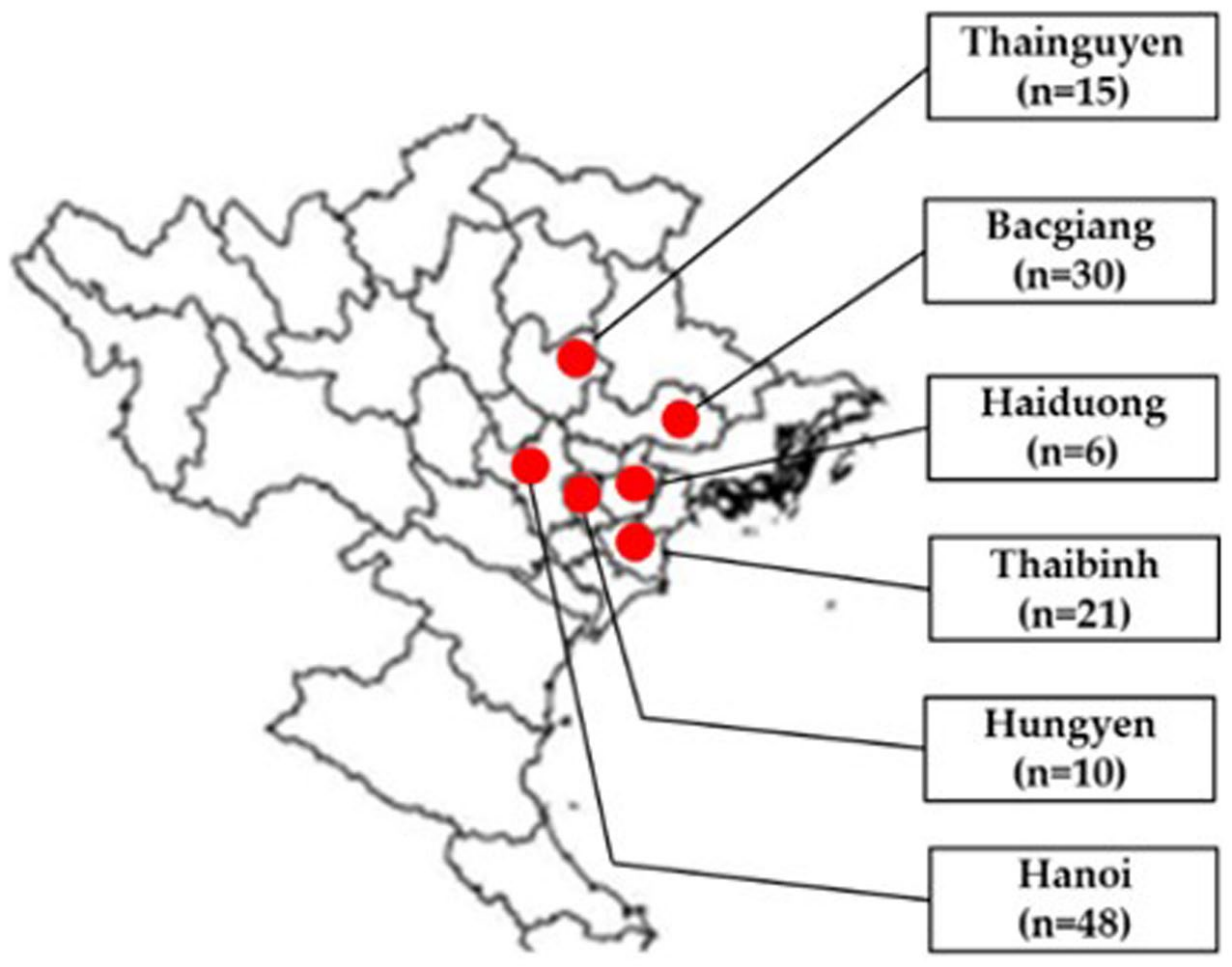
Location of sampling areas in north Vietnam in provinces/cities (red circles): Thainguyen (*n* = 15), Bacgiang (*n* = 30), Haiduong (*n* = 6), Thaibinh (*n* = 21), Hungyen (*n* = 10), and Hanoi (*n* = 48). Adapted from Dong et al. (12); licensed under CC BY 4.0.

In total, 130 tissue samples (brain, lung, liver, kidney and bursa of Fabricius) of broiler ducks aged 2–7 weeks were collected from Bacgiang, Haiduong, Hanoi, Hungyen, Thaibinh and Thainguyen in 2021 (Figure 1). In each flock, 2–6 diseased ducks were collected by local veterinarians, necropsied, packed with dry ice, and transported to the Vietnam National University of Agriculture for laboratory analysis. Tissue samples of each duck were pooled for further testing. A 10% homogenization of pooled samples was prepared in phosphate-buffered saline.

### 2.3 RNA extraction, cDNA synthesis and conventional PCR for TMUV detection

A Viral Gene-spin^™^ Viral DNA/RNA Extraction Kit (iNtRON Biotechnology; Seoul, Korea) was used for the RNA extraction from homogenized samples, following the manufacturer’s instruction. M-MLV enzyme (Invitrogen; Carlsbad, CA, United States) was used to synthesize cDNA. In total, 20 μL of reagents, consisting of 4 μL of 5X M-MLV buffer, 1 μL of dNTP, 1 μL of random primer (Invitrogen; Carlsbad, CA, United States), 1 μL of MMLV reverse transcriptase (Invitrogen; Carlsbad, CA, United States), and 9 μL of distilled water, were mixed with 4 μL of RNA. Then, the mixture was placed in the thermal machine at 25°C for 10 min, 37°C for 1 h, and 65°C for 10 min.

Identification of the TMUV genome was carried out using PCR to detect the target 400 bp gene, with the TV-3F and TV-3R primers (Table 1), as described elsewhere (13). PCR was performed using GoTag^™^ Green Master Mix (Promega) at 94°C for 5 min, 40 cycles of 94°C for 30 s, then 55°C for 30 s and 72°C for 30 s, with an extension step at 72°C for 10 min. The PCR products were loaded in 1.5% agarose gels for electrophoresis and then the gel was photographed under UV light.

**Table 1:** Primers used in this study.

Primer	Nucleotide sequence (5′-3′)	PCR product (bp)	Reference
TV-3F	GCCACGGAATTAGCGGTTGT	400	(1)
TV-3R	TAATCCTCCATCTCAGCGGTGTAG		
E-F	CTTGTTTGGAAAGGGRAGYATA	923	(2)
E-R	CTTTKAGTGTTGACGTGAARGC		
prM-F	CGCGGATCCCTGAAGCTTGGAAATTAT	501	(3)
prM-R	GGAATTCGCTGTACGCTGGGGCTATT		

### 2.4 Nucleotide sequencing

Two pairs of primers (E-F/R and prM-F/R; Table 1) were used for amplification of the partial prM and E gene sequences of the TMUV strains. The thermal conditions were 94°C for 4 min, followed by 35 cycles of 94°C for 60 s, then 50°C for 60 s and 72°C for 60 s, with a final extension step at 72°C for 5 min. The PCR products were loaded in 1.5% agarose gels for electrophoresis. The PCR products were purified using GeneClean^®^ II Kits (MP Biomedicals; Santa Ana, CA, United States). Sequencing of the TMUV strains was performed by 1st BASE (Malaysia).

### 2.5 Genetic and phylogenetic analyses

The nucleotide sequences from the Vietnamese TMUV strains identified in this study were aligned using the Bioedit software supplemented with Clustal W (14, 15). Nucleotide identity among the Vietnamese and other sequences downloaded in GenBank were identified using the Basic Local Alignment Search Tool (BLAST)^1^ and the GENETYX version 10.0 software (GENETYX Corp.; Tokyo, Japan). In total, 40 and 38 sequences of the E and prM genes, respectively, from different TMUV strains from GenBank (Table 2) were used to construct phylogenetic trees and further conduct genetic characterization of the viral strains. Maximum likelihood methods, based on the Tamura 2-parameter model, were used to established phylogenetic trees. The MEGA X^2^ software was used with bootstrapping of 1,000 replicates to determine the confidence values of tree branches. The partial E and partial prM gene sequences obtained were deposited into GenBank, with the accession numbers OR727885 to OR727894, respectively.

**Table 2:** Description of Tembusu virus strains used in this study.

GenBank accession No.	Strain	Location	Source	Year
NC_015843.2	JS804/2010	China	Goose	2010
MN649264.1	GX2012	China	Duck	2012
MF581314.1	JX14	China	Goose	2014
KX686575.1	HD2	China	Duck	2013
KX686579.1	HZ3	China	Duck	2015
KY623441.1	JS201502	China	Duck	2015
KY623440.1	ZJ201508	China	Duck	2015
KY623439.1	ZJ201506	China	Duck	2015
KY623436.1	ZJ201503	China	Duck	2015
KM275940.1	GX2013E	China	Duck	2013
MF522174.1	zjYY150901	China	Duck	2015
KY623433.1	zjYY150902	China	Duck	2015
MF581312.1	GX15	China	Goose	2015
KX686580.1	HZ-2014	China	Duck	2014
KJ700462.1	GX2013H	China	Duck	2013
KM275941.1	GX2013G	China	Duck	2013
KM233707.1	CQW1	China	Duck	2013
MK542820.1	Y	China	Duck	2019
KP742476.1	TMUV-SH001	China	Duck	2015
KJ740748.1	AHQY	China	Duck	2013
KF826767.	TMUV-SDHS	China	*Passer domesticus*	2012
JX273153.1	JS	China	Duck	2010
MN649267.1	AH2014	China	Duck	2014
KU323595.1	GD2014	China	Muscovy duck	2014
MH748542.1	SD14	China	Duck	2014
KX686577.1	HD1	China	Duck	2013
KX686572.1	HD	China	Duck	2015
MN649266.1	HB2016	China	Duck	2016
MZ031023.1	JXSP-310	China	Duck	2017
KC810847.1	AFRIMS-D119-014/150	Thailand	Duck	2002
MK276427.1	DK/TH/CU-56	Thailand	Duck	2016
KC810846.1	JSL385	Thailand	Mosquito	2002
KR061333.1	DK/TH/CU-1	Thailand	Duck	2013
MK276420.1	DK/TH/CU-18	Thailand	Duck	2015
MK276442.1	DK/TH/CU-130	Thailand	Duck	2016
KF573582.1	KPS54A61/THA	Thailand	Duck	2013
AB110495.1	ThCar105/92	Malaysia	Duck	2016
JX477685.2	MM_1775	Malaysia	Duck	2012
JX477686.1	Sitiawan virus	Malaysia	Duck	2012
MN747003.2	TP1906	Taiwan	Mosquito	2019

### 2.6 Analyses of recombination and natural selection profiles

The selected TMUV strains obtained in this study and other sequences from GenBank were used to examine recombination events based on the Recombination Detection Program (RDP; version Beta 4.97), containing the RDP, GENECONV, BootScan, MaxChi, Chimera, SiScan, Phyl-Pro, LARD, and 3Seq methods, with default settings (13). Evaluation of natural selection profiles was performed using the FUBAR (a Fast Unconstrained Bayesian AppRoximation) method (16).^3^

### 2.7 Statistical analysis

Significant differences in the rate of the TMUV genome between geographical regions, ages, or flock size groups were detected using Fisher’s exact test. A value of *p* < 0.05 was used to determine significant differences.

## 3 Results

### 3.1 Identification of TMUV genome in field samples

In this study, 21 (16.15%) out of 130 samples were detected as positive for the TMUV genome based on the PCR method. Detailed information of positive samples was indicated in Supplementary Table 1. The TMUV-positive rates were 30 and 26.67% in Bac Giang and Thai Nguyen, respectively, which were significantly greater than for Haiduong (16.67%), Hanoi (10.42%), Hungyen (10%), and Thaibinh (4.76%). Of the 38 tested duck farms, 9 (23.68%) were positive for the viral genome. Among the cities/provinces, the highest positive rate was 50% in Hai Duong, which was significantly higher than for Thainguyen (33.33%), Bacgiang (27.27%), Hungyen (25%), Hanoi (18.18%), and Thaibinh (14.29%), as shown in Table 3.

**Table 3:** Identification of Tembusu virus in individual ducks and farms from different locations in north Vietnam.

Province/ City	Individual level			Farm level		
	No. of samples	Gene-positive samples	Positive rate (%)	No. of farms	Gene-positive flocks	Positive rate (%)
Hanoi	48	5	10.42^a^	11	2	18.18^c^
Haiduong	6	1	16.67^a^	2	1	50.00^a^
Thainguyen	15	4	26.67^ab^	3	1	33.33^b^
Bacgiang	30	9	30.00^b^	11	3	27.27^b^
Thaibinh	21	1	4.76^ac^	7	1	14.29^c^
Hungyen	10	1	10.00^a^	4	1	25.00^b^
Total	130	21	16.15	38	9	23.68

^a, b, and c^values with different lowercase superscripts in a column are significantly (*p* < 0.05) different.

The percentage of TMUV-positive ducklings at age 2–4 weeks was 59.26%, which was significantly higher than for those aged >4 weeks (5.43%), while there were no positive samples from ducks aged <2 weeks for the viral genome using the PCR method. In this study, the farm levels were divided into three levels (1 with the number of ducklings <500, 2 with the number of ducks in the range 500–1,000, and 3 with the number of ducks >1,000 ducks). The positive rates were 17.24, 18.75, and 10.81% for levels 1, 2, and 3 flocks; however, these rates were not significantly different (Table 4).

**Table 4:** Detection of Tembusu virus genome in field samples based on age and flock size.

Criteria	Level	No. of samples	No. of gene-positive samples/(%)
Age (weeks)	< 2	11	0/(0)
	2–4	27	16/(59.26)^a^
	> 4	92	5/(5.43)^b^
Flock size (number of ducks)	<500	29	5/(17.24)
	500–1,000	64	12/(18.75)
	>1,000	37	4/(10.81)

^a and b^age groups with different lowercase superscripts are significantly (*p* < 0.05) different.

### 3.2 Genetic and phylogenetic characterization of Vietnamese TMUV strains

Among the TMUV-positive samples, five were randomly selected for sequencing of the partial E and prM genes of the viral strains. The Vietnamese TMUV strains obtained in this study were designated as Vietnam/VNUA-28, −31, −101, −102, and 117/2021. Regarding the partial E gene (891 bp) sequences, the nucleotide identity ranged from 99.55% (VNUA-101/2021 vs. VNUA-102/2021) to 100% (VNUA-28/2021 vs. VNUA-101/2021). The Vietnamese nucleotide identities were from 94.51 to 94.73% compared to the vaccine strain (China/JXSP-310/2017), as shown in Table 5.

**Table 5:** Nucleotide identities of partial E (891 bp) gene sequences among Vietnamese and vaccine TMUV strains.

Strain name	Strain name					
	Nucleotide identity (%)					
	VNUA-28/2021	VNUA-31/2021	VNUA-101/2021	VNUA-102/2021	VNUA-117/2021	China/ JXSP-310/2017
VNUA-28/2021	100.0					
VNUA-31/2021	99.66	100.0				
VNUA-101/2021	100.0	99.66	100.0			
VNUA-102/2021	99.55	99.21	99.55	100.0		
VNUA-117/2021	99.66	99.32	99.66	99.88	100.0	
China/JXSP-310/2017	94.73	94.51	94.73	94.51	94.62	100.0

In terms of the prM gene (498 bp), the nucleotide identity ranged from 99.59% (VNUA-28/2021 vs. VNUA-31/2021) to 100% (VNUA-102/2021 vs. VNUA-117/2021). Comparison with the vaccine strain indicated that the nucleotide identities were from 93.57 to 93.97% (Table 6).

**Table 6:** Nucleotide identities of partial prM gene sequences (498 bp) among Vietnamese and vaccine TMUV strains.

Strain name	Strain name					
	Nucleotide identity (%)					
	VNUA-28/2021	VNUA-31/2021	VNUA-101/2021	VNUA-102/2021	VNUA-117/2021	China/JXSP-310/2017
VNUA-28/2021	100.0					
VNUA-31/2021	99.59	100.0				
VNUA-101/2021	99.79	99.79	100.0			
VNUA-102/2021	99.79	99.79	99.59	100.0		
VNUA-117/2021	99.79	99.79	99.59	100.0	100.0	
China/JXSP-310/2017	93.77	93.77	93.97	93.57	93.57	100.0

In addition, the five Vietnamese TMUV strains were compared with sequences from abroad downloaded from GenBank. These results indicated that the highest nucleotide identities for the partial E gene sequences were 96.52% (China/GX2012 vs. VNUA-31/2021), 96.64% (China/GX2013H VNUA-102; China/GX2012 vs. VNUA-117/2021), and 96.75% (China/GX2012 vs. VNUA-28 and -101/2021) for the partial E gene sequences, while the highest nucleotide identities for the partial prM gene sequences were 96.38% (China/SD201120 vs. VNUA-28/2021), 96.58% (China/SD201120 vs. VNUA-101, −102, −117/2021), and 96.78% (China/SD201120 vs. VNUA-31/2021), as shown in Table 7.

**Table 7:** Nucleotide identities of partial E (891 bp) and prM (498 bp) gene sequences between Vietnamese TMUV strains and viruses retrieved from GenBank.

	Virus with highest nucleotide identity							
Strain name	Partial E gene				Partial prM gene			
	Accession number	Strain	Country	% identity	Accession number	Strain name	Country	% identity
VNUA-28/2021	MN649264.1	GX2012	China	96.75	KY623423.1	SD201120	China	96.38
VNUA-31/2021	MN649264.1	GX2012	China	96.52	KY623423.1	SD201120	China	96.78
VNUA-101/2021	MN649264.1	GX2012	China	96.75	KY623423.1	SD201120	China	96.58
VNUA-102/2021	KJ700462.1	GX2013H	China	96.64	KY623423.1	SD201120	China	96.58
VNUA-117/2021	MN649264.1	GX2012	China	96.64	KY623423.1	SD201120	China	96.58

**Figure 2:**
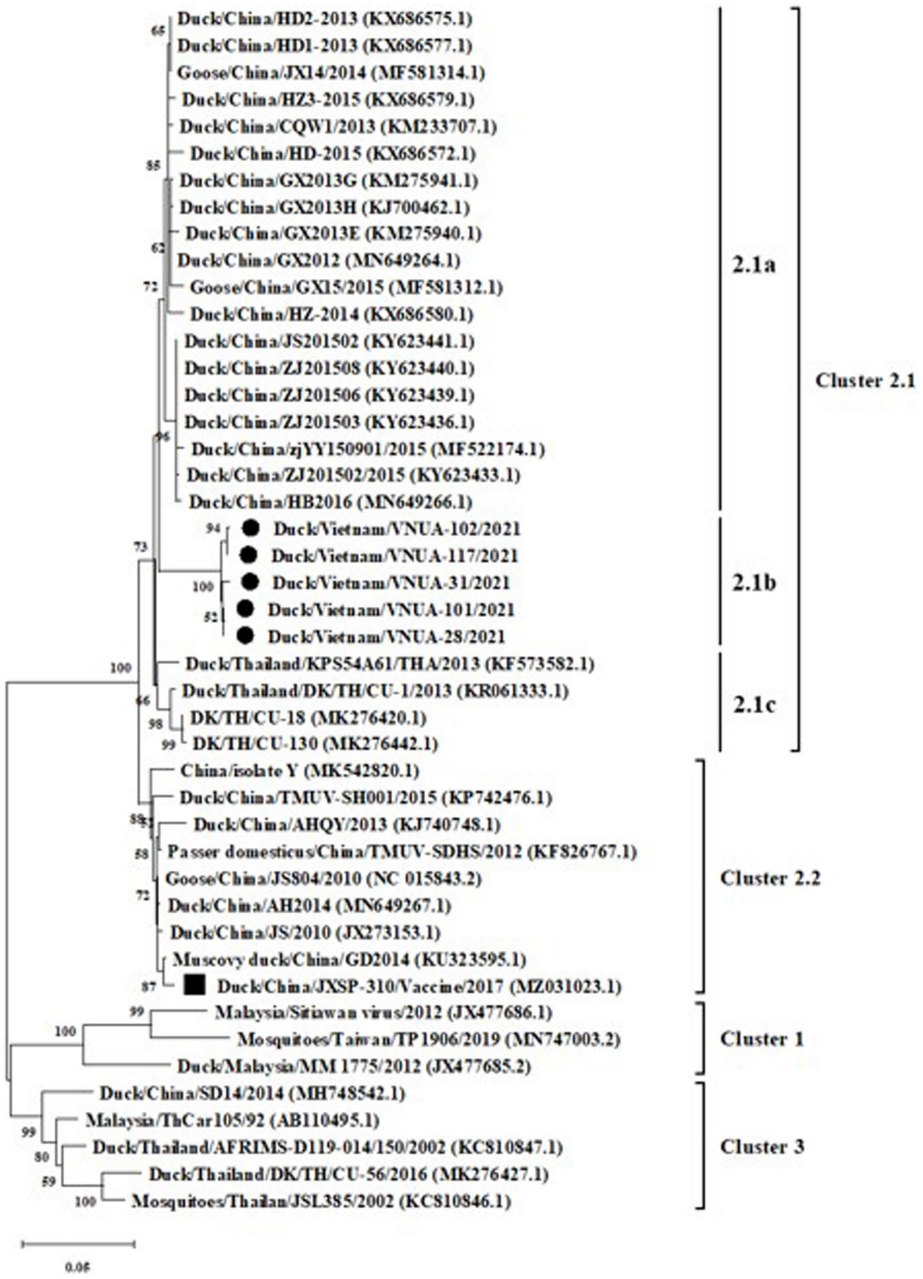
A Maximum likelihood phylogenetic tree of partial E gene (891 bp) sequences of Vietnamese Tembusu virus strains compared with those available in GenBank. GenBank sequences are indicated by country name/accession number. The maximum likelihood method in the MEGA X software was used to establish phylogenetic trees (1,000 bootstrap replicates). Numbers at each branch point indicated bootstrap values ≥50% in the bootstrap interior branch test. The current Vietnamese strains are indicated by black-filled circles, while the vaccine strain is marked by a black-filled square.

**Figure 3:**
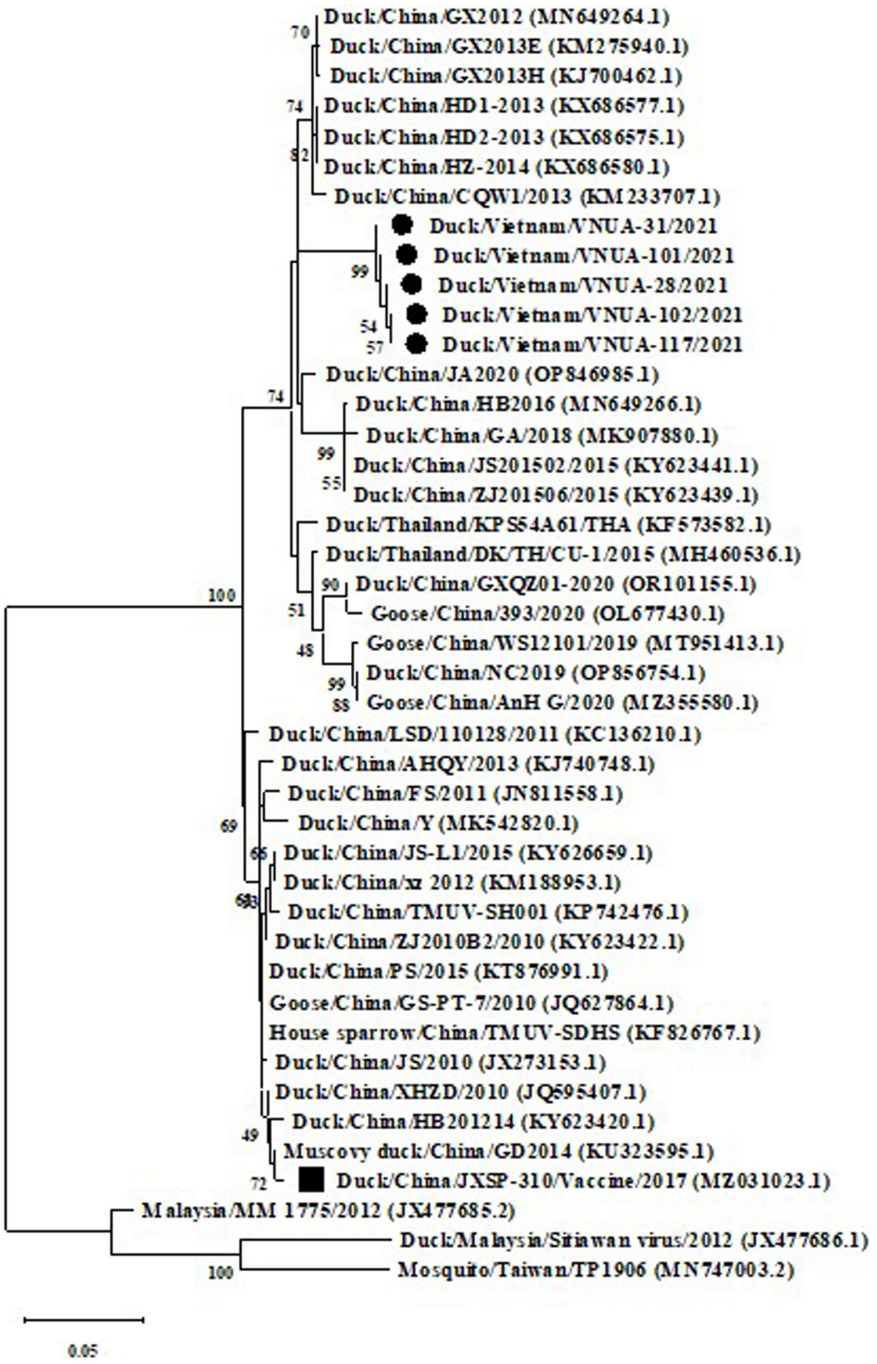
A Maximum likelihood phylogenetic trees of partial prM gene (498 bp) sequences of Vietnamese Tembusu virus strains compared with those available in GenBank. GenBank sequences are indicated by country name/accession number. The maximum likelihood method in the MEGA X software was used to establish phylogenetic trees (1,000 bootstrap replicates). Numbers at each branch point indicated bootstrap values ≥50% in the bootstrap interior branch test. The current Vietnamese strains are indicated by black-filled circles, while the vaccine strain is marked by a black-filled square.

Phylogenetic trees were established based on the partial E (891 bp) and prM (498 bp) sequences of the five TMUVs and other viruses from GenBank. The results indicated that the five Vietnamese TMUV strains obtained in this study formed a novel single cluster (2.1b). The five Vietnamese TMUV strains were genetically related to Chinese strains and differed from the vaccine strain China/JXSP-310/2017 (MZ031023.1), as shown in Figures 2, 3.

Among the five Vietnamese TMUV strains, the deduced amino acid (aa) of the partial E protein indicated that there were three major substitutions, consisting of 110 K/E, 157A/V, and 345D/N. Substitution at the 345 residue was unique, in the Vietnam/VNUA-102 and -117/2021 strains, D → N. For the vaccine strain (China/JXSP-310/2017), there were 11 aa substitutions detected at residues 110, 122, 157, 176, 196, 277, 314, 345, 367, 391, and 394 (Table 8).

**Table 8:** Amino acid substitutions of E protein among Vietnamese TMUV strains compared with vaccine strain.

Virus strain	Virus strain										
	110	122	157	176	196	277	314	345	367	391	394
Consensus^a^	K	T	A	Y	V	N	N	D	T	E	K
Vietnam/VNUA-28/2021	.^b^	A	.	.	.	.	.	.	.	.	.
Vietnam/VNUA-31/2021	.	A	V	.	.	.	.	.	.	.	.
Vietnam/VNUA-101/2021	.	A	.	.	.	.	.	.	.	.	.
Vietnam/VNUA-102/2021	E	A	.	.	.	.	.	N	.	.	.
Vietnam/VNUA-117/2021	.	A	.	.	.	.	.	N	.	.	.
China/JXSP-310/2017	K	.	.	H	A	S	S	.	K	G	R

^a^The consensus sequence was released with 100 partial E gene sequences of TMUV strains from GenBank by the GENETYX software. ^b^ same as the consensus sequence.

No recombination event was found among the Vietnamese TMUVs obtained in this study based on the partial E gene sequences. The selection profiles of the five obtained TMUV strains and other strains from GenBank were analyzed based on the partial E protein (297 amino acids). These results indicated that 169 sites had negative selection (Supplementary Table 2), whereas no positive selection was found on the partial E protein of the Vietnamese TMUV strains.

## 4 Discussion

It has been reported that TMUV was successfully isolated from mosquitoes in Malaysia in 1950s (17). Subsequently, the virus spread and has been reported in many countries globally (1, 2, 4, 7, 11, 18). Understanding infection is important in creating strategies to control the disease. In Vietnam, the first report of TMUV infection was conducted in 2019 (11). The current study has continued to describe viral infection among ducks raised in some provinces in north Vietnam. Epidemiological analysis was first conducted in this study, with the five novel Vietnamese TMUV strains obtained in this study genetically forming a single cluster (2b), separated from the vaccine strain based on the partial E gene sequences (891 bp). This finding suggested that these strains were field strains.

Another study noted that TMUV infection could reach 90% among ducks within a farm, while the mortality rate varied from 5 to 30% (19). In the current study, The rates for TMUV-positive samples and farms were 16.15 and 23.68%, respectively, based on PCR. These rates were lower than the rate of 46.59% (41/88) reported in China during 2010–2016 (20) due to the fact that the sampling number, location, and time parameters were different, resulting in differences in the infection rates. Li et al. conducted a study to access pathogenicity of TMUV strains isolated in China (21). One-, 3-, and 7-weeks-old ducks were inoculated with TMUV strains. The study pointed out that ducks at one-week-old indicated severe symptoms, while other groups showed milder pathological lesions (21). This finding agreed with a previous study revealed that young ducks were more sensitive to TMUV strains than older ducks, suggesting a relationship between viral susceptibility and age (21, 22). Later researchers found that ducks aged 18–21 weeks and 55 weeks were susceptible to the disease caused by TMUV (23, 24). Changes in age-related susceptibility occurred among ducks infected with TMUV strains. It is due to the fact that host immune responses, different viral strains, or evolution among viral strains may contribute to age-related susceptibility. Further studies should be conducted to elucidate these points. The current study indicated that the highest rate of viral infection was in young ducks aged 2–4 weeks (59.26%), compared to older ducks aged >4 weeks (5.43%), with no positive samples being detected in ducks aged <2 weeks. Older ducks may have a stronger immune response, leading to lower positive rates (21). Further studies should be conducted to explain the relationship between age and infection rates in north Vietnam.

Genetic and phylogenetic analyses based on the partial E and prM gene sequences revealed that the current five Vietnamese TMUV strains formed a novel subcluster, which was closely related to Chinese strains. The results suggested that the TMUV strains obtained in this study might have a similar origin to Chinese strains. N-linked glycosylation, which contributes to the entry of the virus into host cells, was predicted in the E protein of TMUV at the 103, 154, and 314 residues (25, 26). However, in the current study, substitutions were not found at these three residues, suggesting the conservation of the glycosylation sites of E protein among Vietnamese TMUV strains, which was similar to the findings of Huang et al. Fritz et al. suggested that protonation of histidine’s plays an important role in the membrane fusion of flaviviruses (27). No substitutions of histidine was observed at the 144, 153, 163, 219, 246, 263, 285, 320, and 398 residues of the E protein of the five Vietnamese TMUVs in the current study, nor were substitutions found in the epitopes 220–226 and the 374–380 residues, located in domains DII and DIII in the E protein of the Vietnamese TMUV strains. In the current study, three substitutions were found among Vietnamese TMUV strains: residues 110 (K → E), 157 (A → V), and 345 (D → N). To our knowledge, no evidence on the contribution of these substitutions has been reported; other studies will be conducted to evaluate this point.

One of the evolutionary processes is recombination, was first found among TMUV strains in China (28). The current study did not find any recombination events among the Vietnamese TMUV strains based on analysis of the partial S gene. Expanding analysis of the complete genome and increasing the number of sequences are necessary to examine possible recombination events among Vietnamese TMUV strains. Dai et al. (16) reported that negative selection was strong among Chinese TMUV strains. A few positive selection sites were found among the TMUV strains as a result of genetic drifts, similar to other flaviviruses (29, 30). In the current study, 169 residues of Vietnamese TMUV E protein had negative selection.

## 5 Conclusion

In this study, TMUV infection in samples and on duck farms were 16.15 and 23.68%, respectively, in six provinces/city in north Vietnam in 2021. The infection was most commonly detected in young ducks aged 2–4 weeks (59.26%), at a significantly higher level than in ducks aged <2 weeks and > 4 weeks. Genetic and phylogenetic analyses of the five Vietnamese TMUV strains based on the partial E and prM gene sequences supported that the current Vietnamese TMUV strains belonged to a novel subcluster, which was closely related to the Chinese strains and differed from the vaccine strain. No putative recombination event was detected among the Vietnamese TMUV strains. Strong negative selection was found among the Vietnamese TMUV strains, based on the analysis of the partial E protein. Further studies should be conducted to better understand the evaluation of TMUV strains across the country.

## Data availability statement

The original contributions presented in the study are publicly available. This data can be found here: https://www.ncbi.nlm.nih.gov/nuccore/; OR727885-OR727894.

## Ethics statement

The animal studies were approved by the Committee on Animal Research and Ethics of the University (CARE-2021/04). The studies were conducted in accordance with the local legislation and institutional requirements. Written informed consent was obtained from the owners for the participation of their animals in this study.

## Author contributions

HD: Conceptualization, Methodology, Software, Writing – original draft, Data curation, Formal analysis, Investigation, Visualization. GT: Conceptualization, Writing – original draft, Data curation, Formal analysis, Investigation, Visualization. TV: Writing – original draft. NL: Writing – original draft. YN: Writing – original draft. WR: Writing – original draft. AR: Writing – original draft. CB: Writing – original draft. DB: Writing – original draft, Conceptualization, Software, Supervision, Writing – review & editing. JR: Conceptualization, Funding acquisition, Methodology, Project administration, Resources, Software, Supervision, Validation, Writing – original draft, Writing – review & editing.

## Footnotes

^1^ https://blast.ncbi.nlm.nih.gov/

^2^ https://megasoftware.net/

^3^ http://www.datamonkey.org/
