# Supplementary material for: Duck Tembusu virus in North Vietnam: epidemiological and genetic analysis reveals novel virus strains
Source: Front Vet Sci. 2024 May 14;11:1366904. doi: 10.3389/fvets.2024.1366904 (PMC11134369; doi:10.3389/fvets.2024.1366904)
Supplement: Supplementary file 1 [file Table_1.DOC]

Supplemental Table 1. Natural selection profile in partial E protein of four current Vietnamese Tembusu virus strains and other strains from GenBank

| Site | α | β | β - α | Prob  [α > β] | Prob  [α < β] | Bayes Factor  [α < β] |
| --- | --- | --- | --- | --- | --- | --- |
|
| 102 | 2.735 | 0.197 | -2.539 | 0.936 | 0.05 | 0.143 |
| 103 | 3.119 | 0.151 | -2.968 | 0.977 | 0.017 | 0.047 |
| 104 | 4.018 | 0.145 | -3.873 | 0.975 | 0.019 | 0.054 |
| 105 | 5.102 | 0.235 | -4.866 | 0.985 | 0.01 | 0.028 |
| 106 | 2.590 | 0.165 | -2.425 | 0.945 | 0.043 | 0.122 |
| 107 | 3.404 | 0.146 | -3.258 | 0.984 | 0.011 | 0.032 |
| 108 | 2.810 | 0.137 | -2.673 | 0.975 | 0.018 | 0.051 |
| 110 | 3.407 | 0.122 | -3.284 | 0.988 | 0.009 | 0.024 |
| 111 | 3.124 | 0.168 | -2.955 | 0.972 | 0.021 | 0.057 |
| 112 | 2.316 | 0.195 | -2.121 | 0.923 | 0.06 | 0.174 |
| 115 | 2.807 | 0.203 | -2.603 | 0.935 | 0.051 | 0.147 |
| 116 | 2.586 | 0.17 | -2.416 | 0.943 | 0.045 | 0.127 |
| 117 | 2.716 | 0.209 | -2.508 | 0.93 | 0.055 | 0.159 |
| 120 | 5.614 | 0.163 | -5.451 | 0.999 | 0 | 0.001 |
| 121 | 1.190 | 0.273 | -0.917 | 0.728 | 0.225 | 0.791 |
| 122 | 7.696 | 0.29 | -7.405 | 0.996 | 0.002 | 0.005 |
| 123 | 1.195 | 0.323 | -0.871 | 0.697 | 0.256 | 0.936 |
| 124 | 2.739 | 0.208 | -2.531 | 0.931 | 0.055 | 0.157 |
| 125 | 8.225 | 0.251 | -7.974 | 0.999 | 0 | 0.001 |
| 126 | 0.581 | 0.644 | 0.063 | 0.379 | 0.56 | 3.472 |
| 127 | 2.619 | 0.25 | -2.369 | 0.912 | 0.07 | 0.205 |
| 128 | 2.011 | 0.183 | -1.828 | 0.917 | 0.065 | 0.188 |
| 129 | 2.807 | 0.207 | -2.600 | 0.933 | 0.053 | 0.151 |
| 130 | 3.445 | 0.134 | -3.311 | 0.986 | 0.01 | 0.027 |
| 131 | 2.586 | 0.17 | -2.416 | 0.943 | 0.045 | 0.127 |
| 132 | 3.134 | 0.136 | -2.998 | 0.98 | 0.015 | 0.04 |
| 133 | 4.886 | 0.14 | -4.746 | 0.997 | 0.002 | 0.004 |
| 136 | 2.082 | 0.222 | -1.860 | 0.902 | 0.078 | 0.23 |
| 137 | 3.842 | 0.123 | -3.719 | 0.996 | 0.003 | 0.008 |
| 138 | 5.73 | 0.172 | -5.557 | 0.998 | 0.001 | 0.003 |
| 139 | 3.049 | 0.208 | -2.841 | 0.937 | 0.049 | 0.141 |
| 143 | 2.20 | 0.22 | -1.980 | 0.906 | 0.074 | 0.218 |
| 144 | 6.914 | 0.571 | -6.342 | 0.973 | 0.017 | 0.047 |
| 145 | 0.599 | 0.742 | 0.143 | 0.352 | 0.589 | 3.895 |
| 146 | 5.923 | 0.208 | -5.715 | 0.999 | 0.001 | 0.002 |
| 151 | 2.572 | 0.165 | -2.407 | 0.944 | 0.043 | 0.123 |
| 152 | 2.059 | 0.201 | -1.858 | 0.932 | 0.052 | 0.149 |
| 153 | 6.524 | 0.682 | -5.842 | 0.932 | 0.046 | 0.133 |
| 154 | 2.06 | 0.192 | -1.868 | 0.914 | 0.067 | 0.196 |
| 155 | 6.937 | 0.206 | -6.731 | 1 | 0 | 0.001 |
| 156 | 3.654 | 0.125 | -3.529 | 0.994 | 0.004 | 0.01 |
| 158 | 5.846 | 0.162 | -5.685 | 0.999 | 0 | 0.001 |
| 159 | 2.421 | 0.174 | -2.247 | 0.936 | 0.05 | 0.143 |
| 160 | 5.622 | 0.157 | -5.465 | 0.98 | 0.015 | 0.042 |
| 161 | 2.475 | 0.168 | -2.307 | 0.94 | 0.046 | 0.133 |
| 162 | 2.32 | 0.148 | -2.172 | 0.945 | 0.042 | 0.12 |
| 163 | 2.726 | 0.205 | -2.522 | 0.932 | 0.054 | 0.155 |
| 165 | 3.7 | 0.104 | -3.596 | 0.997 | 0.002 | 0.006 |
| 166 | 2.881 | 0.135 | -2.747 | 0.977 | 0.017 | 0.047 |
| 167 | 5.911 | 0.656 | -5.256 | 0.962 | 0.021 | 0.058 |
| 168 | 6.243 | 0.176 | -6.067 | 0.999 | 0 | 0.001 |
| 170 | 4.225 | 0.185 | -4.041 | 0.985 | 0.01 | 0.029 |
| 175 | 3.913 | 0.154 | -3.759 | 0.993 | 0.004 | 0.012 |
| 178 | 2.473 | 0.169 | -2.305 | 0.94 | 0.047 | 0.133 |
| 179 | 2.825 | 0.11 | -2.715 | 0.982 | 0.013 | 0.035 |
| 180 | 2.805 | 0.207 | -2.598 | 0.933 | 0.052 | 0.151 |
| 181 | 3.036 | 0.151 | -2.885 | 0.975 | 0.018 | 0.051 |
| 186 | 3.725 | 0.166 | -3.559 | 0.983 | 0.012 | 0.033 |
| 190 | 2.463 | 0.205 | -2.258 | 0.924 | 0.06 | 0.173 |
| 192 | 8.169 | 0.217 | -7.952 | 1 | 0 | 0 |
| 194 | 1.891 | 0.205 | -1.686 | 0.901 | 0.078 | 0.23 |
| 195 | 4.523 | 0.134 | -4.389 | 0.997 | 0.002 | 0.005 |
| 196 | 1.990 | 0.194 | -1.796 | 0.911 | 0.07 | 0.205 |
| 198 | 2.481 | 0.205 | -2.276 | 0.923 | 0.06 | 0.175 |
| 200 | 4.669 | 0.189 | -4.480 | 0.988 | 0.009 | 0.024 |
| 204 | 1.935 | 0.202 | -1.733 | 0.904 | 0.075 | 0.221 |
| 205 | 6.161 | 0.213 | -5.948 | 0.999 | 0.001 | 0.001 |
| 209 | 4.175 | 0.125 | -4.050 | 0.996 | 0.002 | 0.007 |
| 213 | 2.949 | 0.162 | -2.787 | 0.971 | 0.021 | 0.059 |
| 218 | 2.501 | 0.204 | -2.298 | 0.926 | 0.058 | 0.169 |
| 221 | 3.759 | 0.14 | -3.618 | 0.988 | 0.009 | 0.024 |
| 222 | 2.436 | 0.206 | -2.229 | 0.922 | 0.061 | 0.177 |
| 223 | 2.572 | 0.166 | -2.406 | 0.944 | 0.043 | 0.124 |
| 226 | 3.105 | 0.163 | -2.942 | 0.973 | 0.02 | 0.055 |
| 229 | 2.499 | 0.168 | -2.331 | 0.941 | 0.046 | 0.131 |
| 230 | 3.518 | 0.141 | -3.378 | 0.992 | 0.006 | 0.015 |
| 232 | 2.849 | 0.207 | -2.643 | 0.935 | 0.051 | 0.147 |
| 233 | 5.067 | 0.148 | -4.920 | 0.999 | 0.001 | 0.002 |
| 234 | 2.564 | 0.215 | -2.349 | 0.923 | 0.061 | 0.176 |
| 235 | 2.642 | 0.21 | -2.432 | 0.927 | 0.057 | 0.165 |
| 236 | 2.799 | 0.138 | -2.662 | 0.974 | 0.019 | 0.052 |
| 237 | 4.987 | 0.153 | -4.835 | 0.997 | 0.002 | 0.005 |
| 238 | 3.147 | 0.135 | -3.012 | 0.981 | 0.014 | 0.038 |
| 239 | 1.897 | 0.202 | -1.695 | 0.902 | 0.077 | 0.226 |
| 241 | 2.79 | 0.246 | -2.544 | 0.919 | 0.064 | 0.187 |
| 242 | 5.435 | 0.166 | -5.269 | 0.998 | 0.001 | 0.003 |
| 243 | 2.472 | 0.171 | -2.301 | 0.939 | 0.048 | 0.136 |
| 244 | 2.479 | 0.172 | -2.308 | 0.939 | 0.048 | 0.136 |
| 245 | 1.946 | 0.202 | -1.744 | 0.905 | 0.075 | 0.219 |
| 246 | 2.648 | 0.161 | -2.487 | 0.964 | 0.026 | 0.073 |
| 247 | 3.934 | 0.144 | -3.790 | 0.989 | 0.008 | 0.022 |
| 248 | 2.59 | 0.165 | -2.425 | 0.945 | 0.043 | 0.122 |
| 249 | 7.456 | 0.281 | -7.175 | 0.996 | 0.002 | 0.005 |
| 250 | 2.729 | 0.208 | -2.521 | 0.93 | 0.055 | 0.157 |
| 253 | 1.912 | 0.172 | -1.740 | 0.92 | 0.062 | 0.18 |
| 255 | 5.668 | 0.584 | -5.084 | 0.948 | 0.037 | 0.104 |
| 258 | 8.396 | 0.233 | -8.163 | 1 | 0 | 0 |
| 260 | 3.597 | 0.123 | -3.474 | 0.994 | 0.004 | 0.01 |
| 262 | 3.671 | 0.114 | -3.557 | 0.991 | 0.006 | 0.017 |
| 263 | 2.48 | 0.171 | -2.309 | 0.939 | 0.047 | 0.136 |
| 265 | 4.651 | 0.174 | -4.477 | 0.995 | 0.003 | 0.009 |
| 266 | 1.921 | 0.195 | -1.726 | 0.907 | 0.072 | 0.213 |
| 267 | 3.885 | 0.17 | -3.715 | 0.984 | 0.011 | 0.031 |
| 268 | 13.177 | 1.134 | -12.044 | 0.99 | 0.001 | 0.003 |
| 269 | 2.619 | 0.208 | -2.412 | 0.928 | 0.057 | 0.164 |
| 270 | 2.843 | 0.132 | -2.711 | 0.984 | 0.011 | 0.029 |
| 275 | 5.272 | 0.190 | -5.082 | 0.998 | 0.001 | 0.002 |
| 276 | 2.104 | 0.188 | -1.916 | 0.918 | 0.064 | 0.186 |
| 277 | 3.778 | 0.107 | -3.671 | 0.997 | 0.002 | 0.006 |
| 279 | 3.055 | 0.151 | -2.904 | 0.975 | 0.018 | 0.05 |
| 280 | 2.568 | 0.195 | -2.374 | 0.931 | 0.054 | 0.155 |
| 281 | 3.410 | 0.134 | -3.276 | 0.986 | 0.01 | 0.028 |
| 284 | 2.561 | 0.255 | -2.305 | 0.907 | 0.074 | 0.216 |
| 286 | 3.371 | 0.137 | -3.234 | 0.991 | 0.006 | 0.017 |
| 287 | 2.731 | 0.206 | -2.525 | 0.932 | 0.053 | 0.154 |
| 288 | 2.228 | 0.185 | -2.044 | 0.944 | 0.042 | 0.119 |
| 289 | 2.726 | 0.205 | -2.522 | 0.932 | 0.054 | 0.155 |
| 292 | 1.990 | 0.194 | -1.796 | 0.911 | 0.07 | 0.205 |
| 293 | 3.390 | 0.164 | -3.226 | 0.978 | 0.016 | 0.044 |
| 294 | 4.009 | 0.120 | -3.889 | 0.993 | 0.005 | 0.014 |
| 296 | 4.125 | 0.149 | -3.977 | 0.995 | 0.003 | 0.009 |
| 298 | 5.821 | 0.203 | -5.618 | 0.999 | 0.001 | 0.002 |
| 299 | 2.510 | 0.168 | -2.343 | 0.941 | 0.046 | 0.13 |
| 300 | 4.002 | 0.122 | -3.881 | 0.996 | 0.002 | 0.006 |
| 301 | 2.849 | 0.129 | -2.72 | 0.978 | 0.016 | 0.045 |
| 302 | 2.024 | 0.205 | -1.819 | 0.929 | 0.054 | 0.156 |
| 303 | 8.664 | 0.592 | -8.072 | 0.992 | 0.004 | 0.011 |
| 306 | 3.102 | 0.111 | -2.991 | 0.986 | 0.01 | 0.029 |
| 307 | 2.792 | 0.133 | -2.659 | 0.976 | 0.018 | 0.049 |
| 310 | 5.637 | 0.202 | -5.435 | 0.997 | 0.002 | 0.005 |
| 313 | 3.419 | 0.131 | -3.287 | 0.986 | 0.01 | 0.027 |
| 314 | 3.109 | 0.132 | -2.976 | 0.982 | 0.013 | 0.037 |
| 315 | 2.518 | 0.173 | -2.345 | 0.939 | 0.048 | 0.136 |
| 317 | 2.739 | 0.208 | -2.531 | 0.931 | 0.055 | 0.157 |
| 321 | 2.586 | 0.17 | -2.416 | 0.943 | 0.045 | 0.127 |
| 326 | 3.053 | 0.109 | -2.944 | 0.986 | 0.01 | 0.028 |
| 327 | 2.971 | 0.149 | -2.822 | 0.975 | 0.018 | 0.05 |
| 329 | 2.789 | 0.136 | -2.653 | 0.975 | 0.019 | 0.051 |
| 330 | 2.889 | 0.111 | -2.778 | 0.983 | 0.012 | 0.033 |
| 331 | 4.508 | 0.155 | -4.353 | 0.969 | 0.023 | 0.065 |
| 334 | 2.484 | 0.167 | -2.316 | 0.941 | 0.046 | 0.131 |
| 335 | 6.085 | 0.183 | -5.902 | 0.998 | 0.001 | 0.003 |
| 337 | 3.050 | 0.131 | -2.919 | 0.988 | 0.008 | 0.022 |
| 339 | 3.053 | 0.164 | -2.889 | 0.973 | 0.02 | 0.054 |
| 342 | 2.525 | 0.141 | -2.384 | 0.953 | 0.036 | 0.101 |
| 344 | 3.809 | 0.169 | -3.641 | 0.984 | 0.012 | 0.032 |
| 345 | 2.881 | 0.128 | -2.752 | 0.979 | 0.015 | 0.042 |
| 346 | 2.907 | 0.132 | -2.775 | 0.986 | 0.01 | 0.027 |
| 349 | 2.881 | 0.135 | -2.746 | 0.977 | 0.017 | 0.047 |
| 351 | 2.520 | 0.141 | -2.379 | 0.953 | 0.036 | 0.102 |
| 352 | 4.864 | 0.18 | -4.684 | 0.995 | 0.003 | 0.008 |
| 353 | 2.469 | 0.172 | -2.297 | 0.938 | 0.048 | 0.137 |
| 354 | 2.476 | 0.168 | -2.308 | 0.94 | 0.046 | 0.132 |
| 358 | 7.957 | 0.869 | -7.088 | 0.969 | 0.014 | 0.039 |
| 362 | 10.308 | 0.565 | -9.743 | 0.992 | 0.005 | 0.013 |
| 364 | 2.472 | 0.171 | -2.301 | 0.939 | 0.048 | 0.136 |
| 365 | 4.309 | 0.185 | -4.124 | 0.986 | 0.01 | 0.028 |
| 369 | 2.600 | 0.139 | -2.461 | 0.956 | 0.034 | 0.095 |
| 371 | 2.436 | 0.206 | -2.229 | 0.922 | 0.061 | 0.177 |
| 374 | 4.74 | 0.138 | -4.602 | 0.997 | 0.002 | 0.005 |
| 375 | 3.244 | 0.129 | -3.115 | 0.984 | 0.011 | 0.032 |
| 376 | 3.695 | 0.134 | -3.561 | 0.997 | 0.002 | 0.005 |
| 377 | 4.452 | 0.599 | -3.852 | 0.917 | 0.059 | 0.171 |
| 378 | 6.954 | 0.227 | -6.727 | 0.998 | 0.001 | 0.004 |
| 380 | 3.791 | 0.168 | -3.622 | 0.984 | 0.012 | 0.032 |
| 384 | 4.267 | 0.134 | -4.133 | 0.997 | 0.002 | 0.006 |
| 389 | 4.289 | 0.146 | -4.143 | 0.992 | 0.005 | 0.015 |
| 393 | 5.074 | 0.171 | -4.902 | 0.996 | 0.002 | 0.006 |
| 396 | 2.510 | 0.206 | -2.304 | 0.924 | 0.059 | 0.172 |
